# Topics of Public Interest

**Published:** 1917-01

**Authors:** 


### TOPICS OF PUBLIC INTEREST

**Location for Physician.** An admirable office suite, long used by a physician recently deceased, is vacant. This is a good opportunity for a young physician. Inquire of the editor before 1 p. m.

**Influence of War on Canadian Universities.** Arts Departments of Toronto, University College, Victoria, Trinity and St. Michael's give a total registration of 1215, against 1853 in 1915-16, 2161 in 1914-15, 1574 in 1913-14, a gradual reduction to less than 50% of the normal. Applied Science: 192 in 1916-17, 345 in 1915-16, 563 in 1914-15, 627 in 1913-14, a gradual reduction to about 30% of the normal. Forestry: 10 in 1916-17, 32 in 1915-16, 48 in 1914-15, 51 in 1913-14, a reduction to 20%. Medicine: 399 in 1916-17, 614 in 1915-16, 660 in 1914-15, 623 in 1913-14, a reduction to about 65%. Undergraduates and graduates of the University of Toronto, serving under the colors, number at present 3020, and over 140 have died.

**Canadian Casualties.** Out of a total of 59,911 reported to Oct. 31, 1916, prisoners not being included unless counted as presumably dead or missing, 9,457 were killed in action, 3,477 died of wounds, 490 of sickness, 1,027 are presumed to be dead, and 2,245 are reported as missing. Wounded but not dead 43,245. It will be noted that the ratio of killed to wounded is about 1:5, about as for previous wars, though statements have been made that the proportion of killed has been much higher in the present war. Until quite recently in military history, it has been accepted almost as an axiom that deaths from sickness and from wounds are about equal. The very low mortality from disease shows how great advances have been made by military medicine—yet, it is not recognized as a distinct branch. 1,501 cases of typhoid have occurred: 903 among inoculated and 508 among uninoculated, though we understand that the intention has been to inoculate practically all. There were 166 typhoid deaths, rather more than the 10% expected on the average, but not excessive in consideration of the unfavorable factors in military environment. 47 deaths occurred among the 903 cases in inoculated soldiers—5.2%; 119 among the 508 cases in uninoculated soldiers—23.4%. Presumably the antis will note merely that a majority of cases occurred among the uninoculated. 2,118 paratyphoid cases occurred with 29 deaths showing that this is by no means a mild infection but quite as serious as typhoid. 1,968 occurred among inoculated with

22 deaths, almost exactly 11½; and 150 among uninoculated, with 7 deaths, about 5%. The moral here is not so plain.

---

**Rabies.** Dr. Anderson C. Crowforth, a veterinarian of Lockport, was bitten in the face by a mad dog (diagnosis verified by Cornell University laboratory) about Dec. 15. He received inoculation at the Pasteur Institute of N. Y. Several cases in dogs are reported from near Bergholz, and one in a cow. A man was bitten but he will receive Pasteur treatment. No human cases are reported from Buffalo but a considerable number among dogs. Note: As the influence of a hydrophobia scare is bad in itself, and as many persons are opposed to quarantine, there is need of popular education on the subject. Even from the professional standpoint, it is desirable that reports should be as accurate as possible, and that they should be stated in terms of actual evidence. There are a good many problems to be solved in connection with this disease, both diagnostic and therapeutic, and the utmost care should be taken to avoid false impressions in either direction.)

---

**Six-Year Medical Course.** The University of Toronto has decided to lengthen the course to six years, after July, 1918. The first year will be really premedical or collegiate, with amplified courses in physics, chemistry and biology. We understand also that the fifth year at present, or the sixth year under the new requirement, is really a compulsory interneship. Thus the change is not so radical as at first appears.

---

**Football Casualties.** 15 lives were lost this year as against 16 last year. Probably the deaths from exposure among the onlookers exceed these numbers. Football will become safe when, like baseball, every play will be made without interference. On such a basis would not football be a more brilliant and interesting game, as well as safer?

---

**A Series of Attempts at Criminal Assault** are reported from Niagara Falls, various philanthropic institutions being asked by telephone to send a woman on an errand of mercy of some sort, to a desolate locality. In one case, a nurse from the Memorial Hospital was attacked but fought off her assailant. In another case, the Salvation Army was appealed to, but the man in charge refused to send a woman to attend another woman in distress and offered to go himself, but the sender of the message hung up the telephone. Dec. 21, a nurse was decoyed from N. Tonawanda to the River Road but succeeded in fighting off her assailant. The precautionary measures against such miserable practices are obvious.

**The Genesee County Home** cost, for the fiscal year ending Dec. 1, 44 cents a day per capita. Produce raised on the farm, in addition to that consumed, was sold to the amount of $4,737.

---

**Tuberculosis Hospital Site.** The supervisors of Steuben Co. have voted, 24 to 9, to purchase the Wm. Gelder farm near Bath, for $7,000. The selection was made partly because this farm adjoins that already occupied by the Almshouse—which may be a good point economically, but is open to some theoretic sanitary objection.

---

**Niagara Falls Hospital Addition.** The Memorial Hospital includes a maternity building known as the Louise Memorial, erected at the expense of the late Benjamin F. Thurston. Need has developed for additions to this building, and funds amounting to $30,000 have been donated by Mr. and Mrs. Hugh Gladding of Providence, as a memorial to Mr. Thurston, who was Mrs. Gladding's brother. The present building will be remodeled and two new wings will be added, each 2 stories high, with a basement 28x50 feet. Other improvements will consist in the installation of elevators, storage rooms, private rooms, a signal system, etc.

---

**Increase of Rates by Niagara Falls Physicians.** At a recent meeting of the Academy of Medicine, day rates will be increased from $1.50 to $2.00, and night rates from $2 to $3

---

**Aggregate Versus Specific Mortality.** During the months of June, July and August, N. Y. City had a lower mortality for children under 5 than for many years, in spite of the poliomyelitis epidemic. The various sanitary and hygienic precautions, which proved in vain for the latter, evidently were worth while, on general principles. There is a valuable lesson here, and it is to be hoped that it will not require an epidemic to inforce it in the future, for any city.

---

**Physician Wanted.** The position of Secretary to the Georgia Board of Health is vacant at $2,000 a year. Application should be made to Dr. W. H. Doughty, Jr., Augusta, Ga.

---

**Back Numbers Wanted.** Jan., May, Aug. to Nov. inclusive, 1915. Previous announcement superseded. Buffalo Medical Journal.

---

**The N. Y. State Museum,** partially destroyed by fire several years ago, will be formally opened Dec. 29. The editor acknowledges the courtesy of an invitation.

**Prevalence of Constipation.** The 5 and 10 cent stores seem to be doing a large business in pocket size fountain syringes. Either constipation is resuming Its former prevalence and the laity imagine that it can be relieved by small enemata so frequently repeated as to require portability or, in quite a diff-erent connection, our legislation is burying its head in the sand, to blind itself to the presence of danger—as scientists declare the ostrich does not do.

---

**Tuberculosis Hospitals.** Rensselaer, Livingston and Warren Counties have recently voted affirmatively on this proposition, the respective appropriations being $150,000, $35,000 and $50,000. In all, 12 counties have passed measures of this sort, submitted to popular vote. The aggregate appropriation was $645,000. The total vote was 144,893, of which 87,983 was favorable to the proposition. The average favorable vote of almost exactly two-thirds of the total, is scarcely so high as might be desired, especially when we consider that more than half the voters are not tax-payers, therefore, not voting in behalf of their pocketbooks and that the measure is one that, on its face, would be expected to appeal to the human instincts of the people. While we are inclined strongly to favor the establishment of county hospitals, it should be borne in mind that almost every question referred to popular vote will receive a majority, unless it is plainly objectionable, those who are luke-warm or mildly prejudiced, often failing to vote instead of voting negatively. A two-thirds vote of this nature does not represent a stronger conviction than a 50% vote on a candidate. It is discouragingly small and perhaps explains the small number of counties in which the matter has been the subject of a referendum. The objection of a third of the voters ought to be the subject of careful inquiry. It is merely an unwillingness or inability to assume an additional burden, remembering that at least a quarter of all taxes are for compulsory philanthropy, or its opposition to the commonly accepted professional view as to tuberculosis, or is it due to the conviction that the county hospital does not satisfactorily solve the problem?

---

**Yearly Medical Examination.** The American Society for the Control of Cancer and the National Assn. for the Study and Prevention of Tuberculosis co-operated in an endeavor to educate the public in the importance of anticipating serious developments of disease. By matter distributed for publication on Dec. 4, the assistance of the press was sought to make Dec. 6, a national medical examination day. The editor frankly confesses not to have had a single applicant for examination on this day or within the week, that could in any way be attributed to this agitation. Will our readers kindly contribute their experience? (Answer: They will not, it is too much trouble).

**Prize Competition.** The Fiske Fund trustees offer a prize of $200 for the best essay submitted before May 1, 1917, on The Role of Teeth and Tonsils in the Causation of Arthritis. The ordinary requirements of secresy and typing of Mss. apply. Address Dr. Halsey De Wolf, 305 Brook Street, Providence.

**Infantile Paralysis.** A case developed in a 13-year old boy, 4 miles north of Perry, N. Y., about Dec. 10. This is the first and, thus far, the only case in the vicinity.

**Centenarian.** Asa E. Halbert of Portville, Cattaraugus Co., celebrated his 100th anniversary Dec. 14.

